# Parkinson's Disease and Cognitive Impairment

**DOI:** 10.1155/2016/6734678

**Published:** 2016-12-12

**Authors:** Yang Yang, Bei-sha Tang, Ji-feng Guo

**Affiliations:** ^1^Department of Neurology, Xiangya Hospital, Central South University, Changsha, Hunan 410008, China; ^2^Key Laboratory of Hunan Province in Neurodegenerative Disorders, Central South University, Changsha, Hunan 410008, China; ^3^State Key Laboratory of Medical Genetics, Changsha, Hunan 410008, China; ^4^Neurodegenerative Disorders Research Center, Central South University, Changsha, Hunan 410008, China

## Abstract

Parkinson's disease (PD) is a progressive neurodegenerative disease primarily characterized by the hallmarks of motor symptoms, such as tremor, bradykinesia, rigidity, and postural instability. However, through clinical investigations in patients and experimental findings in animal models of Parkinson's disease for years, it is now well recognized that Parkinson's disease is more than just a motor-deficit disorder. The majority of Parkinson's disease patients suffer from nonmotor disabilities, for instance, cognitive impairment, autonomic dysfunction, sensory dysfunction, and sleep disorder. So far, anti-PD prescriptions and surgical treatments have been mainly focusing on motor dysfunctions, leaving cognitive impairment a marginal clinical field. Within the nonmotor symptoms, cognitive impairment is one of the most common and significant aspects of Parkinson's disease, and cognitive deficits such as dysexecutive syndrome and visuospatial disturbances could seriously affect the quality of life, reduce life expectancy, prolong the duration of hospitalization, and therefore increase burdens of caregiver and medical costs. In this review, we have done a retrospective study of the recent related researches on epidemiology, clinical manifestation and diagnosis, genetics, and potential treatment of cognitive deficits in Parkinson's disease, aiming to provide a summary of cognitive impairment in Parkinson's disease and make it easy for clinicians to tackle this challenging issue in their future practice.

## 1. Introduction

In developed countries, nearly one out of 100 people older than 60 years old are affected by Parkinson's disease [1]. Cognitive impairment in Parkinson's disease, characterized by predominant executive deficits, visuospatial dysfunction, and relatively unaffected memory, ranges from Parkinson's disease mild cognitive impairment (PD-MCI) to Parkinson's disease dementia (PDD), the former of which could only be detected by various means of comprehensive neuropsychological observations and normally does not affect the patients' daily operations whereas the latter hits more than one area of cognition and is severe enough to impair social or working functions. Moreover, longitudinal studies of long-term clinical investigations suggested that the majority of PD or PD-MCI patients develop dementia as disease deteriorates into the late stage [2–4], and Parkinson's disease dementia is a critically influential factor for the reduced life expectancy in patients with Parkinson's disease [5]. Movement disorder has long been addressed to be burdensome in Parkinson's disease and the development of relatively effective restoration of dopamine by pharmaceutical treatment also contributes to the success of management of motor symptoms, leaving the treatment of nonmotor deficits an unmet clinical need. Furthermore, the aggravation of cognitive disturbances might also be strongly predicted by neuropsychological testing in the early stage of disease with or without timely medical treatment [5–8].

In this review, we illustrate the demographic and clinical symptoms potentially assessed as risk factors for nonmotor deficits in Parkinson's disease and discuss the underlying mechanisms of these symptoms with evidence from genetic studies, with primary focus on the clinical manifestations and diagnosis supported by neuropsychology research, neuroimaging, pharmacology, and molecular genetics. At last, we probe into the clinical pharmacological and nonpharmacological management for Parkinson's disease patients in the light of its heterogeneous nature.

## 2. Epidemiology

PD is one of the most common neurodegenerative disorders, whose incidence is second only to Alzheimer disease. According to a 5-year follow-up study by Broeders and a Norwegian ParkWest study by Pedersen, 25% to 50% of patients with Parkinson disease develop PD-MCI or PDD or progress from PD-MCI to PDD within 5 years of diagnosis [9, 10]. Studies that followed patients prospectively diagnosed PD with normal cognition and discovered the incidence of cognitive impairment are few till now. However, according to the available evidence, the progression of cognitive impairment was very common and comparatively quick. For instance, one study exhibited that the cumulative incidence of developing cognitive impairment was 8.5% within 1-year follow-up and up to 47.4% within 6-year follow-up [11]. In other studies, the incidence of cognitive impairments in PD patients varied from 48% to 60% by 12–15 years of retrospective follow-up [12, 13]. In addition, the community-based studies indicated that 20–35% of PD population would develop PD-MCI and up to 10% would develop PDD per year [14, 15]. Nonetheless, it is difficult to compare the results of all studies mentioned above, due to differences in sample sizes and statistical methods used. Furthermore, one designed study also clarified that the onset of dementia in PD patients is approximately 70-year-old no matter when the onset of PD is [16].

Not only does the incidence of cognitive impairment in PD patients vary, but also the risk factors for PD-MCI and PDD vary. Pigott et al. claimed that increased baseline Hoehn & Yahr Scale score and Unified PD Rating Scale motor score, and decreased baseline Dementia Rating Scale (DRS-2) scores are powerful predictors of early cognitive deficits [17]. It is widely accepted that DRS-2 might be effective and adequate for predicting cognitive disturbance and could be used as a reference method to test comprehensive cognitive function [16, 18].

## 3. Etiology

In this part, we mainly focus on the genetics of PD. 18 PD-specific chromosomal loci are named* PARK* and numbered chronologically, nine of which have been identified and confirmed by linkage analysis or exome sequencing [19–33]. Eight of these loci were identified by linkage analysis, functional candidate gene approach or GWAS studies, and are deemed as susceptibility loci as risk factors [34–39]. And still one of them is supposed to be erroneous locus found to be identical with* PARK1* [40]. Within the nine confirmed disease-causing genes,* SNCA*,* LRRK*, and* VPS35* exhibit an autosomal dominant hereditary pattern while other six genes,* Parkin*,* PINK1*,* DJ-1*,* ATP13A2*,* PLA2G6*, and* FBX07*, display an autosomal recessive hereditary pattern. Besides, some other genes, such as* GIGYF2*, were reported to be susceptible to PD with specific variants in different ethnic populations [41]. The mutated genes involved in PD cause brain dysfunction through various molecular mechanisms, including disturbance of presynaptic vesicle recycling and dopamine transmission, toxicity from aggregation of mutant proteins, degeneration of dopaminergic axon in substantia nigra, instability or mislocation of certain kinases, overactivation of ubiquitin kinase activities, and decreased efficiencies of ubiquitin degradation pathways [42–52]. Although only 10–15% of PD cases are familial and studies related to the pathogenic mechanisms on the confirmed disease-causing genes or susceptible loci of PD are far from being complete, the discovery of PD-related genes is a critical step for us to unravel the mysteries behind neurodegeneration in PD. Up to date, there is limited research specifically dedicated to the study of the relationship between the genetic classifications of PD and molecular mechanisms of cognitive impairment in PD. However, some negative results indicated some distinctive genetic features of cognitive decline in PD could be differentiated from other neurodegenerative disorders with cognitive disturbances [53, 54]. Furthermore, the filamentous Lewy body formation could be observed in early onset of PDD carrying SNCA mutations and Dementia with Lewy bodies (DLB) [42, 55], and the aggregation of *α*-synuclein could be detected in substantia nigra as well as cortex in idiopathic PD patients, which suggests that the accumulation of *α*-synuclein could be the presynaptic dysfunction attributed to neuronal toxicity caused by various genetic or nongenetic risk factors. It is also found that the frequency of glucocerebrosidase mutations is increased in postmortem samples from PD patients who had positive *α*-synuclein inclusions [56, 57], and the* BDNF* (Met/Met) homozygotes demonstrate dramatically worse cognitive impairment in PD patients compared to noncarriers [58].

## 4. Clinical Characteristics and Diagnosis

There is dramatic heterogeneity in clinical definition and correlation of cognitive impairment in PD, ranging from mild cognitive impairment to dementia [18, 59, 60]. It has been a long time that the definition and characteristics of PD-MCI and PDD exist as a controversial issue, until the Movement Disorder Society (MDS) took the initiative to conduct a Task Force to systematically review the most representative literatures. They evaluated the incidences and characteristics of PD-MCI, as well as its relationship with dementia and its inclination of progressing to dementia [61]. For PD-MCI, the Movement Disorder Society (MDS) finally selected a total of 8 articles (6 cross-sectional studies and 2 longitudinal studies) from 1156 articles (874 for Parkinson & cognitive impairment and 172 for Parkinson & MCI) [18, 59, 62–67], in which the study design, population studied, methodology for statistical analysis, and criteria for PD-MCI/PDD definition vary considerably. On the other hand, publications related to PDD are much more available than those to PD-MCI. The MDS also reviewed the previous publications of dementia in PD excluding the cases of Dementia with Lewy Bodies (DLB) in terms of the “1-year rule,” characterized the clinical manifestations, and used these results to illustrate the criteria of probable and possible PDD based on the consensus from experts [68].

The criteria of both PD-MCI and PDD are defined by clinical, cognitive, and functional aspects. As more time and effort have been devoted to the study of PDD, the criteria for PDD were established first, which also profoundly influenced the proposed criteria for PD-MCI [68, 69]. Similar to the practicality of diagnosis in PDD criteria, a two-level operational schema on the thorough basis of neuropsychological testing is also applied in PD-MCI criteria [69]. Level I is a practical set which could be utilized easily by physicians and needs no neuropsychological testing from neurological or psychological experts, whereas Level II is documented in much more detail and is more favorable for researchers to conduct longitudinal studies.

In a brief assessment of Level I, clinical diagnosis of PD based on Queen's Square Brain Bank criteria for PD must be established for both PD-MCI and PDD [70, 71]. For PD-MCI, cognitive capability is declined slowly which might be described by caregivers or patients or observed by clinicians from testing results. On the other hand, cognitive impairment caused by the clinical manifestations of parkinsonism other than idiopathic PD, other primary possibilities for cognitive disturbances, and other PD-associated comorbid circumstances that could significantly influence the outcome of cognitive testing should be excluded from PD-MCI [68]. The most important point to differentiate PDD from DLB is that PD symptoms should develop prior to the onset of dementia, which could be obtained by clinicians, gathered from the patient him/herself, informant or follow-up records/past medical history [69]. As PD-MCI is a prestage of PDD and progresses to PDD in most cases, the cognitive deficits scaled by a global cognitive ability test or at least two of neuropsychological tests for the five cognitive domains (to erase the limitation of a single neuropsychological test) in PD-MCI should be subtle on complex functional task and not be sufficient to interfere significantly with functional independence [68]. However, the cognitive impairment, which can be examined by global cognitive ability tests (e.g., MMSE below 26 [72]) and by at least two of the neuropsychological tests (months reversed [73] or seven backward [72], lexical fluency or clock drawing [74], MMSE pentagons [72], and 3-word recall [72]), is supposed to be severe enough to impair daily living activities, which could be assessed by a list of simple tasks. And the cognitive impairment should be assessed without administration of antiparkinsonian drugs and not be attributed to other categories of abnormalities such as autonomic or motor symptoms caused by PDD [69].

Once the diagnosis of cognitive impairment, including PD-MCI or PDD, is established, specifying the subtypes of cognitive deficiency and evaluating the severity of disease are quite beneficial for research, clinical practicing and monitoring, and even standardized pharmacological interventions. For PD-MCI diagnosis by Level II criteria, at least two of neuropsychological tests examining each of the five cognitive domains are recommended by MDS. Performance of patients between 1 and 2 standard deviations (SD) below individual variation adjustment showing predominant impairment or premorbid levels may be demonstrated in PD-MCI. But patients within 1 SD below normalization tested by a serial of neuropsychological measurements or who reported significantly cognitive decline over time are also accredited to diagnose PD-MCI [75]. For PD-MCI subtyping, to differentiate PD-MCI as single or multiple domains, at least two neuropsychological tests in each cognitive domain should be conducted. Impaired performance of two tests in the same one cognitive domain without impairment in other cognitive domains demonstrates the single-domain subtype. On the other hand, impaired performance of at least one test in no less than two cognitive domains indicates the multiple-domain type [76–91]. However, for PDD Level II testing, assessments of severity using quantitative measurements do not have upper limit scores in diagnosis. The goal of Level II testing, for one thing, is to confirm the uncertain PDD diagnosis when the clinical manifestations of cognitive impairment are not obvious or relatively confused. It also serves to depict the individual characteristic of PDD and as an indicator of pharmacological responsiveness. In PDD, there are five cognitive domains involved in Level II testing: global cognitive efficiency, executive functions, memory, instrumental functions, and neuropsychiatric functions, in which executive functions and memory are classified as subcorticofrontal functions and instrumental functions are believed to be cortically mediated [92].

## 5. Treatment

Abnormal activities of various subtypes of neurons have been involved in the cognitive impairment of PD, including the dysregulation of dopaminergic, cholinergic, and probably glutamatergic or noradrenergic neurons [93, 94].

Cholinesterase inhibitors, such as rivastigmine, have been proved beneficial to the improvement of global cognition and clinical manifestations as well as neuropsychiatric testing (especially for attention and executive functioning amelioration) by several large-scale multicenter randomized placebo-controlled trials [95–98]. However, Donepezil, also a cholinesterase inhibitor, was not effective for global cognitive improvement or other neuropsychiatric symptoms in PD-MCI or PDD in a large randomized controlled study [99, 100], although its beneficial effect was reported in some small placebo-controlled studies [99].

Partial NMDA-receptor antagonist has been used as a therapeutic option to treat PD patients with cognitive defects in several placebo-controlled trails [101–104]. However, the results of studies were not consistent or notable; only one trial showed statistical differences in the improvement of global cognition [102], whereas most of trials suggested no pharmacological effects of partial NMDA-receptor antagonist on neuropsychiatric symptoms or improvement of daily life [105].

Atomoxetine, a noradrenergic reuptake inhibitor, and clozapine, an inhibitor of serotonin and dopamine receptors, as well as second-generation tricyclic antidepressant (TCA) nortriptyline and pramipexole, have been shown to be beneficial for the regulation of attention, psychosis, and depression, respectively, by evidence from several placebo-controlled trials [93, 106, 107].

Dysexecutive profile, which is known as the most predominant component of cognitive deficits in PD-MCI and PDD, has been substantiated to be improved with levodopa treatment [6, 93]. Levodopa was found to act on some aspects of cognition such as flexibility and working memory without beneficial changes of other functions like visuospatial recognition, verbal ability, or associative learning [6, 93]. For patients with nondopaminergic antiparkinsonian administration, antagonists of the NMDA-type glutamate receptor, amantadine, for instance, could slow down the progressive transition from PD-MCI to PDD, via increasing dopamine release and blocking dopamine reuptake [108].

Subthalamic deep brain stimulation, which is commonly conducted on PD patients with motor complications that are resistant to antiparkinsonian medication, was claimed to be harmful for semantic and verbal fluency as well as executive profiles by a meta-analysis [109]. In the meantime, this invasive procedure, with the possibility of causing damage to the vital brain regions in charge of advanced cognitive functions, has been related to significant exacerbation of dysexecutive profile that is not observed in most desirable pharmacological treatments [110].

Neuroprotective agents aiming to interrupt *α*-synuclein aggregation or to restore neuronal integrity are currently not available, whereas some cognitive interventions that are helpful in Alzheimer's disease have been identified to have positive results in the early stage of randomized clinical studies [111, 112].

While deep brain stimulation (DBS) is effective for the motor deficits of Parkinson's disease (PD) that is well documented, cognitive and psychiatric benefits and side effects from the subthalamic nucleus (STN) and globus pallidus interna (GPi) DBS for PD are increasingly recognized. On one hand, it has been reported that DBS could significantly improve immediate verbal memory and reduce anxiety symptoms [113]; on the other hand, it is also investigated that certain types of impaired domain such as attention impairment predicted more detrimental results after DBS [114]. Therefore, the improvements of cognitive symptoms from DBS require further studies and warrant the precise cognitive tests that stratify the relative risks and benefits of surgery.

## 6. Conclusion

Cognitive impairment in PD, as in other neurodegenerative diseases, demonstrates the common role of neurodegeneration as well as the PD-featured damage in certain advanced cognitive brain regions accompanied with characterized clinical manifestations. The treatments for cognitive deficits in PD remain limited and inadequate since the disturbances of neuronal network involved in the process are still obscure and elusive. As the population ages, the increasing burden for both patients and caregivers from PD-MCI and PDD makes it urgent to approach to the pathogenic mechanisms and therapeutic targets of cognitive deficits in PD, as well as to research and develop novel pharmacological treatments and other interventions that could potentially be used in PD cognitive impairment.
